# Case Report: Hypercalcemia, subcutaneous fat necrosis and nephrocalcinosis in neonates who undergo therapeutic hypothermia: a not so rare association, with different onset time

**DOI:** 10.3389/fped.2024.1477378

**Published:** 2025-01-15

**Authors:** Domenico Umberto De Rose, Chiara Maddaloni, Guglielmo Salvatori, Francesca Campi, Antonio Gatto, Ludovica Martini, Immacolata Savarese, Iliana Bersani, Graziamaria Ubertini, Francesca Serrao, Simonetta Costa, Annabella Braguglia, Francesca Gallini, Giovanni Vento, Andrea Dotta

**Affiliations:** ^1^ Neonatal Intensive Care Unit, “Bambino Gesù” Children’s Hospital IRCCS, Rome, Italy; ^2^ PhD Course in Microbiology, Immunology, Infectious Diseases, and Transplants (MIMIT), Faculty of Medicine and Surgery, “Tor Vergata” University of Rome, Rome, Italy; ^3^ Institute of Pediatrics, Fondazione Policlinico Universitario “Agostino Gemelli” IRCCS, Rome, Italy; ^4^ Pediatric Endocrinology Unit, “Bambino Gesù” Children’s Hospital IRCCS, Rome, Italy; ^5^ Neonatology Unit, Department of Woman and Child Health and Public Health, Fondazione Policlinico Universitario “Agostino Gemelli” IRCCS, Rome, Italy; ^6^ Neonatal Sub-Intensive Care Unit and Follow-up, “Bambino Gesù” Children’s Hospital IRCCS, Rome, Italy; ^7^ Università Cattolica del Sacro Cuore, Rome, Italy; ^8^ Neonatology Unit, Ospedale Isola Tiberina “Gemelli Isola”, Rome, Italy

**Keywords:** hypercalcemia, therapeutic hypothermia, nephrocalcinosis, neonate, hypoxic-ischemic encephalopathy, critical care medicine

## Abstract

Subcutaneous fat necrosis (SCFN) in newborns is an uncommon and self-limiting non-infectious panniculitis. It can occur in the first weeks of life in full-term newborns with hypoxic-ischemic encephalopathy who underwent therapeutic hypothermia. Hypercalcemia may develop and has been implicated as the cause of several complications as nephrocalcinosis. Hypercalcemia has been previously reported to appear only after resolution of skin lesions. Herein, we report how hypercalcemia can be evident already at diagnosis of subcutaneous fat necrosis after therapeutic hypothermia and can be associated with an early onset developing nephrocalcinosis. We compare two cases of these uncommon findings and review the recent literature.

## 1 Introduction

In newborns, subcutaneous fat necrosis (SCFN) is an uncommon and self-limiting non-infectious panniculitis (1). It typically occurs in full-term newborns in the first weeks of life, following perinatal asphyxia, hypothermia, or obstetrical complications during labor or delivery (2). Hypercalcemia may develop and has been implicated as the cause of severe complications, including nephrocalcinosis, hypertension, vomiting, growth failure, irritability, and even seizures.

Herein, we report two cases of newborns with hypercalcemia and subcutaneous fat necrosis after therapeutic hypothermia (TH) associated with nephrocalcinosis; we discuss these findings with their similarities and differences and review the recent literature.

## 2 Methods

In order to review literature, a PubMed (MEDLINE) database search from 1995 onwards was performed using the medical subject headings “subcutaneous fat necrosis” AND “nephrocalcinosis” AND “newborn”; this search revealed few case reports of subcutaneous fat necrosis and nephrocalcinosis in newborns. The search was limited to English for language; the date last searched was March 1st, 2024. Only papers reporting newborns with SCFN and hypercalcemia after therapeutic hypothermia, followed by nephrocalcinosis, were identified in the search.

## 3 Case presentation

### 3.1 Patient 1

A female term child, born at 39 weeks gestational age to a primigravida mother with gestational diabetes, was delivered by emergency cesarean section because of fetal distress in a level II hospital. The birth weight was 3,850 g (large for gestational age according to the Italian Neonatal Anthropometric Charts (3). Apgar scores were 3, 5, and 7 at 1/5/10 min, respectively. The infant was intubated within 2 min of life and placed on mechanical ventilation for respiratory failure. The cord arterial pH was 6.97, and the base excess was −16.8. The infant was moved to the III-level Neonatal intensive care unit (NICU) of Fondazione Policlinico “A. Gemelli” (Rome, Italy) because of moderate encephalopathy on Sarnat staging to start therapeutic hypothermia within the 6-hour window.

On admission to our NICU, she was cooled to 33.5°C for 72 h according to the national neuroprotective cooling protocol, using whole-body cooling. Blood cultures were drawn, and prophylactic antibiotic therapy was started. She was parenterally nourished during the procedure, and no sequelae were observed. After 72 h of therapeutic hypothermia, gradual rewarming to 37°C was performed; mechanical ventilation was gradually withdrawn. Blood exams were always within normal reference ranges during TH and after rewarming, with no remarks on physical examination. On day 5 of life, some red, painful, non-tender subcutaneous lesions appeared on her back in a wide area up to her bottom. The pain was controlled with acetaminophen.

A clinical diagnosis of SCFN was made. Biopsy of the skin and subcutaneous fat was not done, and SCFN was defined by the presence of characteristic clinical features documented by the examining physician.

**Figure 1:**
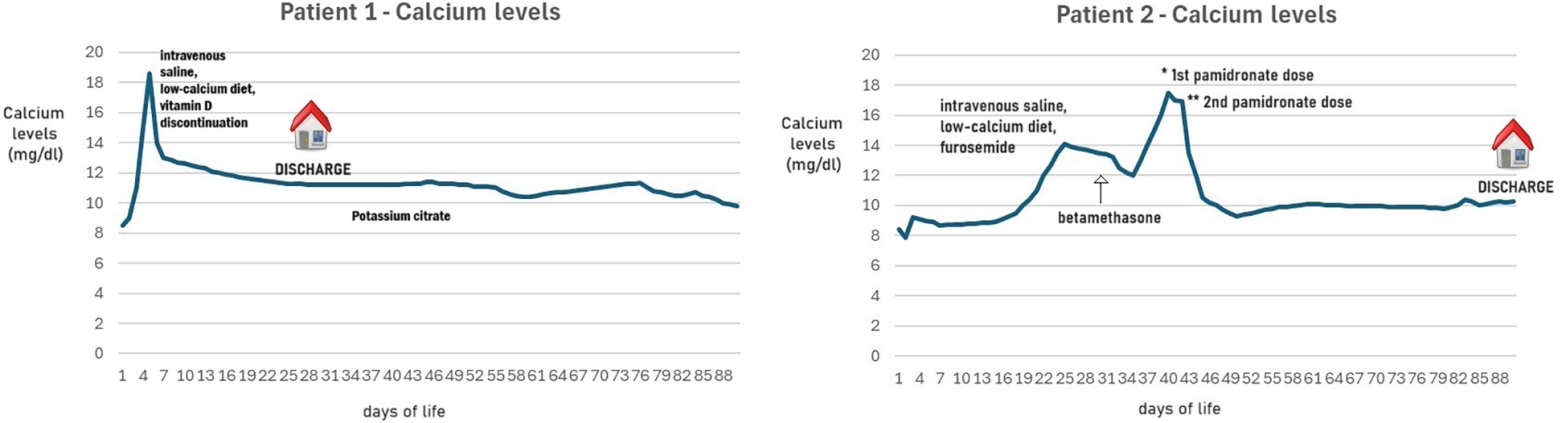
Calcium levels nobserved in patients 1 and 2, treated with TH.

Blood cultures and colliquated area swabs remained negative. A metabolic screening revealed a serum calcium of 18.6 mg/dl (normal range: 8.6–10.2 mg/dl), with a normal creatinine level (Figure 1). This observation led to further tests being performed: serum parathormone by radioimmunoassay was suppressed, and 25-hydroxyvitamin D and 1,25-dihydroxyvitamin D levels were within the normal ranges (Table 1).

**Table 1:** Laboratory data of our patients.

Serum biochemistry test	Patient 1 (maximum value during admission)	Patient 2 (maximum value during admission)	Reference ranges
Calcium	18.6	17.5	8.6–10.2 mg/dl
Phosphorus	1.64 (minimum)	2.30 (minimum)	4.0–7.0 mg/dl
Parathyroid hormone	2.0	2.4	14.0–72.0 pg/ml
25-Hydroxy vitamin D	68.0	31.6	31.0–100.0 ng/ml
1,2-Dihydroxy vitamin D	53.0	NA	16–55 pg/ml
Creatinine	1.0	1.45	0.24–1.0 mg/dl
Urine calcium: creatinine ration	0.13	2.07	<0.6

Considering asymptomatic hypercalcemia, the child was treated with intravenous saline, a low-calcium diet, and vitamin D discontinuation. Neither corticosteroids nor bisphosphonates were used to normalize hypercalcemia.

Magnetic resonance imaging performed on day 5 of life displayed focal brain ischemia in the left basal ganglia, associated with bilateral white matter ischemic spots and cerebral swelling.

The renal ultrasonography showed, at 20 days of age, a bilateral increased echogenicity of medullary pyramids, suggesting a grade 2 nephrocalcinosis secondary to SCFN and hypercalcemia, although renal function was normal.

The child was discharged after twenty-eight days, with a serum calcium level of 11.2 mg/dl. A urine calcium to creatinine ratio of 0.13 (normal: <0.6) excluded hypercalciuria; no evidence of proteinuria was observed.

During follow-up, her serum calcium remained always normal, but considering the persistence of a grade 2 nephrocalcinosis, the child was treated with a potassium citrate supplementation. At 9 months of age, the skin lesions had almost disappeared; however, the renal ultrasonography assessed the persistence of bilateral nephrocalcinosis despite the potassium citrate treatment.

Follow-up visits up to 18 months of age showed normal growth and a mild neurodevelopmental delay, according to brain injury.

### 3.2 Patient 2

A male child, born at 39 weeks gestational age to a tertigravida mother, was delivered by emergency cesarean section because of fetal bradycardia in a level I hospital. The birth weight was 3,220 g (adequate for gestational age according to Italian Neonatal Anthropometric Charts (3). Apgar scores were 1, 1, and 4 at 1/5/10 min, respectively. The infant needed advanced cardiopulmonary support, and he was intubated within 1 min of life and placed on mechanical ventilation for respiratory failure. The cord arterial pH was 6.89, and the base excess was −24. Neurological examination showed a severe encephalopathy on Sarnat staging. The infant was moved to the III-level NICU of Bambino Gesù Children Hospital (Rome, Italy) to start therapeutic hypothermia within the 6-h window.

On admission to our NICU, he was cooled to 33.5°C for 72 h, using whole-body cooling, according to the national neuroprotective cooling protocol. He was in critical condition and required mechanical ventilation and inotropic treatment to maintain hemodynamic stability. Blood cultures were drawn, and empiric antibiotic therapy was started. He was parenterally nourished during the procedure, and no sequelae were observed. At 12 h of life he presented seizures on the amplified electroencephalography (aEEG) and phenobarbital therapy was started.

After 72 h of therapeutic hypothermia, gradual rewarming to 37°C was performed; mechanical ventilation and inotropic therapy were gradually withdrawn and then suspended at 10 days of life. Blood exams were always within normal reference ranges during TH and after rewarming, with no remarks on physical examination.

Magnetic resonance imaging performed on day 7 of life displayed diffuse signal alteration, with hyperintensity in T2-weighted sequences and restriction of diffusivity of the subcortical and deep white matter in the supratentorial hemispheric area bilaterally, of the corpus callosum, of the posterior limb of the internal capsule and of the external capsule bilaterally, of the basal nuclei and of the thalamic region. Similar lesions were evident in the brainstem and the cerebellar cortico-subcortical area bilaterally.

**Figure 2:**
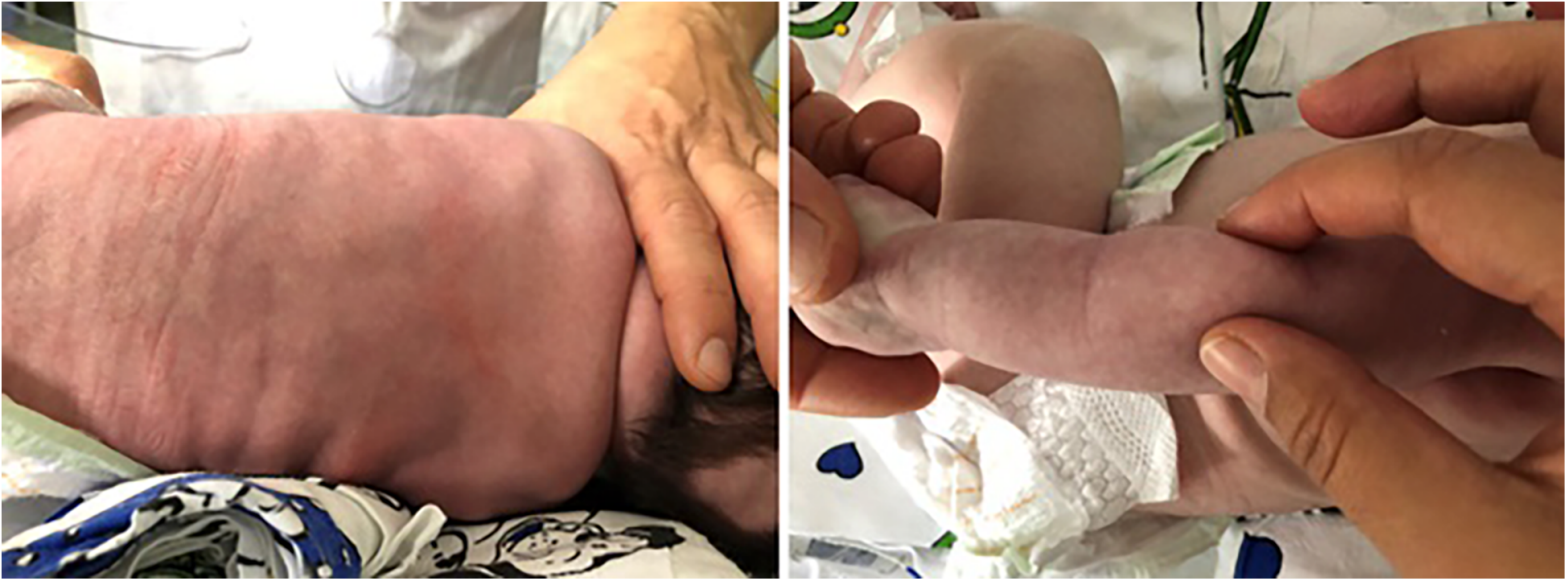
Red, painful, non-tender subcutaneous lesions appeared in patient 2.

On day 25 of life, some red, painful, non-tender subcutaneous lesions appeared on his back in a wide area up to the bottom and on the back of his arms bilaterally (Figure 2). The pain was controlled with acetaminophen.

A clinical diagnosis of SCFN was made. Biopsy of the skin and subcutaneous fat was not done, and SCFN was defined by the presence of characteristic clinical features documented by the examining physician.

A metabolic screening revealed a serum calcium of 14.1 mg/dl (normal range: 8.6–10.2 mg/dl), with a normal creatinine level (Figure 1). This observation led to further tests being performed: serum parathormone by radioimmunoassay was suppressed, and 25-hydroxyvitamin D level was within the normal ranges (Table 1).

The renal ultrasonography, normal at birth, showed a bilaterally increased echogenicity of medullary pyramids, suggesting a grade 2 nephrocalcinosis secondary to SCFN and hypercalcemia, although the renal function was normal.

Considering asymptomatic hypercalcemia, the child was initially treated with hyperhydration of intravenous saline, a low-calcium diet, and furosemide. After a few days, considering the persistence of hypercalcemia and the pain related to the lesions, a course of betamethasone was started with rapid benefit on the skin lesions. Since calcium levels continued to increase (maximum 17.5) at 40 days of life, pamidronate was used in two doses, the first at 0.25 mg/kg and the second at 0.5 mg/kg after 48 h. Calcium levels came back to normal ranges 72 h after the second dose.

The child was discharged at three months of life with a serum level of calcium of 10.3 mg/dl. The skin lesions had disappeared. A urine calcium to creatinine ratio of 0.17 (normal: <0.6) excluded hypercalciuria; no evidence of proteinuria was observed. Considering the presence of grade 2 nephrocalcinosis, the child was treated with a potassium citrate supplementation.

During follow-up, his serum calcium always remained normal, but the last renal ultrasonography, at 12 months of age, assessed the persistence of bilateral nephrocalcinosis despite the potassium citrate treatment.

Follow-up visits up to 24 months of age showed a severe neurodevelopmental delay with bilateral neurosensorial hypoacusis, according to brain injury.

## 4 Results

Sixteen cases were identified (3–11), some of them with incomplete information. In Table 2, we systemically collected and summarized information on patients' characteristics, diagnostic features, laboratory values, duration of symptoms, and evolution under treatment and compared them to our case. Different studies described SCFN and hypercalcemia case reports (10, 12), while we reviewed only the cases of newborns with SCFN, hypercalcemia, and associated nephrocalcinosis after TH.

**Table 2:** Case reports of subcutaneous Fat necrosis and nephrocalcinosis in newborns managed with therapeutic hypothermia.

	Country	Sex	Age at onset of SCFN (days)	Age when SCFN lesions disappeared (months)	Age at onset of hypercalcaemia	Peak serum calcium (mg/dl)	Time to normocalcemia	Age at diagnosis of nephrocalcinosis	Age when nephrocalcinosis disappeared	Treatment	Other symptoms and signs	Reference
1	UK	NA	17	NA	NA	13.5	NA	8 weeks	NA	NA	Vomiting	Strohm 2011 (2)
2	Canada	2 F	10-14	NA	26 days; 29 days	18	13 weeks; 11 weeks	26 days; NA	45 days	PM	NA	Samedi 2014 (4)
5	USA	3 M, 2 F	NA	NA	16-38 days	20.1	2–42 days	NA	Persistent (follow-up of 8–48 months)	IV, F, OC, C, DCR, CIT, PM	Eosinophilia; fever during treatment	Shumer 2014 (5)
3	Canada	NA	10	NA	NA	NA	NA	18 months	24 months	IV, OC, DCR	NA	Del Pozzo-Magaña 2016 (6)
1	Italy	1 F	6	1	5 weeks	NA	3 months	3 months	NA	IV, F	NA	Milanesi 2016 (7)
1	USA	NA	Before 3 weeks	NA	4 weeks	20	7 weeks of life	4 weeks	NA	IV, F, IV, OC, not specified bisphophonate	Vomiting, failure to thrive and irritability	Alsaleem 2019 (8)
1	Turkey	1 F	NA	NA	NA	20	NA	First month of life	NA	PM	NA	Imren, 2020 (9)
1	Greece	1 F	55 days	5 months	55 days	14.2	29 days	NA	NA	IV, F, IC, OC	Poor weight gain and multiple painful subdural nodules	Chrysaidou, 2021 (10)
1	India	1M	7 weeks	NA	7 weeks	20	14 days	7 weeks	NA	IV,F,OC	Lethargy, vomiting, dehydration,	Mehta,2021 (11)
1	Italy	1 F	5	9	5 days	18.6	3 months	20 days	18 months	IV, DCR, CIT	No	Fondazione Policlinico “A.Gemelli” IRCCS, Italy
1	Italy	1 M	25 days	2 months	25 days	17.5	18 days	27 days	NA	IV,F,OC,PM	No	“Bambino Gesù” Children's Hospital IRCCS, Italy

NA, not available; NR, normal range. Treatments abbreviations: C, calcitonin; CIT, citrate; DCR, dietary calcium restriction; F, furosemide; IV, intravenous hydration; IC, intravenous corticosteroids; OC, oral corticosteroids; PM, pamidronate.

## 5 Discussion

The newborn's subcutaneous fat necrosis (SCFN) is an uncommon transient disorder. Usually, it develops within the first weeks of life in full-term newborns who experience perinatal distress, including hypoxic-ischemic encephalopathy (HIE). SCFN is a noninfectious panniculitis characterized by firm subcutaneous nodules, a few millimeters to several centimeters in size, typically found over the face, back, gluteus, arms, and thighs. Resolution of nodules usually takes place over months. These children could present a potentially life-threatening hypercalcemia–hypercalciuria, whose pathogenesis is still unknown. Hypercalcemia, which presents with irritability, lethargy, hypotonia, poor weight gain, and feeding difficulties, must be treated in time to avoid serious complications, such as nephrocalcinosis, nephrolithiasis, and renal failure. Nephrocalcinosis due to SCFN appears after several weeks or months (12).

To the best of our knowledge, the case of patient 1 was interesting because, beyond the rarity of this association, our patient developed SCFN and hypercalcemia early and almost simultaneously. Hypercalcemia has been previously reported to appear only after resolution of skin lesions (4–10). The age of onset of nephrocalcinosis was also early, at 20 days of life, similar to another Canadian clinical report (4). At the time of writing, nephrocalcinosis was still present in our patient at 18 months of life.

Conversely, the case of patient 2 was interesting because the onset of hypercalcemia and SCFN was delayed after the first week of life, but it was prolonged and resistant to the first line of treatment with hyperhydration and diuretic therapy. The calcium levels start to diminish significantly only after pamidronate treatment. In the second case, nephrocalcinosis was evident at the time of diagnosis of SCFN and was still present at the last follow-up at 12 months of age.

Since the introduction of TH for HIE, a few case reports have been published reporting SCFN and nephrocalcinosis. In the United Kingdom, the Total Body Hypothermia (TOBY trial) cooling registry reported incidence of SCFN in 12 of 1,239 (about 1%) newborns who underwent whole-body cooling treatment for HIE; renal ultrasound scan revealed nephrocalcinosis in one of these twelve (about 0.1%) (2). However, the incidence of SCFN and nephrocalcinosis could be underestimated. In our combined multicenter experience, during a six-year period (2018–2023), we have observed 10 cases of SCFN in a multicenter cohort of 315 HIE newborns (about 3.2%), of whom only 2/10 cases with associated nephrocalcinosis (about 0.6%).

Many authors tried to explain the still unknown pathogenesis of SCFN in newborns with perinatal asphyxia. Impaired tissue perfusion and hypoxemia seem to lead to the crystallization of free fatty acids, with subcutaneous adipose tissue storage of them; as a result, tissue necrosis occurs (13). Some authors suggested that the development of SCFN is related to the biochemical characteristics of neonatal adipose tissue: a wrong ratio of unsaturated fatty acid to saturated fatty acids (such as stearic and palmitic acids) permits a setting in which higher melting and solidification points produce fat crystals and tissue necrosis (14). In cold stress conditions such as hypothermia, neonatal adipose tissue is more susceptible to injury, resulting in necrosis of the skin and granulomatous infiltration (13). The differential diagnoses of SCFN include sclerema neonatorum, erythema nodosum, and bacterial cellulitis (4). Bao et al. summarized in a systematic review that patients with SCFN rarely required surgical management: surgery was indicated when the lesions were hemorrhagic, necrotic, or digressed from an ordinary presentation (resembling an infection or a malignancy) (15).

Common complications associated with SCFN include hypoglycemia, anemia, thrombocytopenia, hypertriglyceridemia, and hypercalcemia. In most SCFN cases reported in the literature, hypercalcemia was reported when skin lesions began to resolve. However, there is little data regarding its clinical course: hypercalcemia was mild and follow-up short (16).

Shumer et al. chose to study infants with severe hypercalcemia, probably the ones at the highest risk of complications, in the largest cohort described to date (5). However, few cases reported the association of SCFN with hypocalcemia (17), but the pathogenesis is unknown; perinatal asphyxia may lead to transient pseudohypoparathyroidism and, ultimately, hypocalcemia (18).

Hypercalcemia is the most serious complication of SCFN and usually occurs between one to six months after the fat necrosis is resolved (16, 19). Severe hypercalcemia is rare, but it should be treated immediately and aggressively to avoid serious complications; in particular, nephrocalcinosis is a common complication. In Shumer's cohort, nephrocalcinosis was present in 5/7 patients (83%) with SCFN and hypercalcemia (5).

Khedr et al. reported a case of occult massive visceral fat necrosis following therapeutic hypothermia in a term infant who expired on the 25th day of life following a neonatal course complicated by severe encephalopathy, pulmonary artery hypertension, persistent thrombocytopenia, hypoglycemia, and severe basal ganglia-thalamic abnormalities on magnetic resonance imaging. The postmortem examination indicated extensive necrosis of fat adjacent to ribs, thymus, kidneys, and pancreas, characterized by widespread adipocyte necrosis, granulomatous inflammation, and scattered microcalcifications. Subcutaneous (white) fat was spared. This case highlights the need for close monitoring of encephalopathic newborns for fat necrosis complications, such as hypercalcemia and nephrocalcinosis, as less severe cases may go unnoticed in hypoxic-ischemic newborns, particularly those treated with hypothermia (20).

Concerning the therapeutic approach, the classic treatment of hypercalcemia includes intravenous hyperhydration and restriction of vitamin D and calcium. Indeed, vitamin D supplementation should be avoided (21). However, this first-line therapy could be insufficient and does not change the natural course of the disease and its complications. So, calcium-wasting diuretics (furosemide) and corticosteroids (prednisolone) are used; however, it was described that they increase renal calcium excretion, raising the risk of nephrocalcinosis (22).

Despite the increasing use of bisphosphonates in children for the treatment of primary and secondary forms of osteoporosis and hypercalcemic disorders, inhibiting activity of osteoclasts and bone resorption, few cases of SCFN and hypercalcemia treated with bisphosphonates are reported (23–25). We suggest discussing the possibility of using bisphosphonates when the first-line therapy fails. Pamidronate has been reported as the first-line treatment for severe hypercalcemia, with hypercalciuria complicating SCFN to prevent and decrease the risk of nephrocalcinosis (21). Because it reduces the renal calcium load, it does not increase the risk of nephrocalcinosis as instead corticosteroids and furosemide do (26, 27).

Militello et al. (28) reported a case of a full-term newborn, admitted at 3 weeks of life for failure to thrive and poor feeding, who had at birth a mild perinatal asphyxia that needed no TH (therefore not included in Table 2). Her serum calcium level was 16.6 mg/dl: a renal ultrasound detected bilateral medullary nephrocalcinosis and only later developed SCFN. After intravenous hydration and treatment with furosemide and then intravenous methylprednisolone, they administered a single low dose of zoledronic acid (0.025 mg/kg), and subsequently, the serum calcium rapidly decreased, subcutaneous nodules progressively decreased, and renal findings improved over time.

The use of bisphosphonates in pediatric patients has been proven safe; however, the risk of possible side effects should be kept in mind: flu-like syndrome, hypocalcemia, atypical femur fractures, osteonecrosis of the jaws, orbital inflammation, and growth impairment (25). Their use in newborns should be carefully weighed because there is still insufficient safety data. In our patient, pamidronate was given after unsuccessfully using the classic treatment regimens. Since patient 2 already had nephrocalcinosis, we preferred to avoid long-term diuretic or steroid medication that would have aggravated the renal status, and we observed no side effects after pamidronate administration.

We are aware that the small sample size, with only two cases of SCFN with associated nephrocalcinosis among a multicenter cohort of 315 HIE infants, limits the possibility of generalizing these findings; furthermore, the retrospective nature of this case series introduces potential biases in data collection. Moreover, although HT appeared to have a protective role towards the kidney, as systematically summarized by van Wincoop et al. (29), the follow-up duration, particularly for nephrocalcinosis, may not be sufficient to assess long-term renal outcomes. Indeed, Frank et al. reported that nephrocalcinosis could also be found years later among infants with SCFN (however, including children with SCFN from different causes) (30).

From the review of the literature and the analysis of our case, we suggest strictly monitoring HIE infants who underwent TH with a clinical and ultrasound follow-up, screening them for SCFN and mainly for hypercalcemia already at the onset of skin lesions and also for the possible development of nephrocalcinosis (12). After discharge, parents of infants treated with TH should be, therefore, instructed about all complications and to consult a physician if any symptom of hypercalcemia develops. Even if initially negative, it could be useful to schedule a renal ultrasound again, for example, 3–6 months later, in case of SCFN and/or hypercalcemia during hospitalization.

## 6 Conclusion

Although the association among SCFN, hypercalcemia, and nephrocalcinosis is uncommon, it could be potentially harmful to infants who underwent TH. A multi-disciplinary approach (neonatologist, pediatric endocrinologist, and nephrologist) is required to tailor therapeutic interventions (31). Further studies are needed to evaluate the real incidence of SCFN, hypercalcemia, and nephrocalcinosis in these newborns and standardize the treatment of hypercalcemia to prevent complications.

We suggest that all these infants should be screened for these findings to be treated promptly: our study may help contribute towards a higher level of awareness of this uncommon association.

## Data Availability

The original contributions presented in the study are included in the article/Supplementary Material, further inquiries can be directed to the corresponding author.
